# A novel anti-lipopolysaccharide factor from blue swimmer crab *Portunus pelagicus* and its cytotoxic effect on the prokaryotic expression host, *E. coli* on heterologous expression

**DOI:** 10.1186/s43141-023-00478-w

**Published:** 2023-02-20

**Authors:** M. V. Anju, K. Archana, V. V. Anooja, P. P. Athira, S. Neelima, I. S. Bright Singh, Rosamma Philip

**Affiliations:** 1 Department of Marine Biology, Microbiology and Biochemistry, School of Marine Sciences, Cochin University of Science and Technology, Fine Arts Avenue, Kochi, Kerala 682016 India; 2 National Centre for Aquatic Animal Health, Cochin University of Science and Technology, Kochi, Kerala 682016 India

**Keywords:** Anti-lipopolysaccharide factor, *Portunus pelagicus*, Crustacean immunity, Cytotoxicity, Antimicrobial peptides

## Abstract

**Background:**

Invertebrates like crabs employ their own immune systems to fight against a number of invasive infections. Anti-lipopolysaccharide factors (ALFs) are an important class of antimicrobial peptides (AMPs) exhibiting binding and neutralizing activities against lipopolysaccharides.

**Results:**

This study identified and characterized a novel homolog of ALF (*Pp*-ALF) from the blue swimmer crab *Portunus pelagicus*. *Pp*-ALF has a 369bp open-reading frame encoding a protein with 123 amino acids. The deduced protein featured an LPS-binding domain and a signal peptide. The predicted tertiary structure of *Pp*-ALF contains three *α* helices packed against four *β* sheets. The deduced amino acid sequence of *Pp*-ALF had a net positive charge of +10.75 and an isoelectric point of 9.8. Phylogenetic analysis revealed that *Pp*-ALF has a strong ancestral relationship with crab ALFs.

**Conclusion:**

Antibacterial, antiviral, antifungal, anticancer, and antibiofilm activities of *Pp*-ALF could be revealed by *in silico* prediction tools. Recombinant expression of *Pp*-ALF was unsuccessful in the *Escherichia coli* Rosetta-gami expression system due to the cytotoxic effect of the peptide to the host. The toxic effect of *Pp*-ALF to the host was displayed by membrane permeabilization and death of the host cells by fluorescent staining with Syto9-Propidium Iodide and CTC-DAPI- FITC.

## Background

Emerging issues with antibiotic resistance in microbial pathogens have become a serious threat to the health sector [1]. It is difficult to prevent antibiotic resistance, since it results from adaptation, besides being a fundamental aspect of microbial evolution [2]. Therefore, novel molecules are required as alternatives to antibiotics. Since AMPs operate concurrently on various target sites, they are less likely to cause resistance development compared to antibiotics. They are typically short cationic molecules that are found throughout the entire living kingdom and have a wide range of antibacterial, antifungal, antiviral, and antiparasitic actions [3].

In crustaceans, anti-lipopolysaccharide factors (ALFs) are considered as important antimicrobial peptides that bind to lipopolysaccharides inhibiting the microorganisms [4]. Furthermore, ALFs mediate hemoglobin granulation and the intracellular coagulation cascade [5, 6]. The first ALF was discovered in horse shoe crab, *Limulus polyphemus* [7]. Numerous ALF homologs have now been described in a wide variety of crustacean taxa, including shrimps [8, 4, 9, 10], cray fishes [11], lobsters [12], and crabs [13, 14]. Different isoforms of ALFs have been found in crustacean species; there is very little overlap between the same or related species [15]. Eleven isoforms of ALFs have been identified in *Penaeus monodon* with antibacterial, antifungal, and antiviral functions [16].

Technology for the production of AMPs is developing quickly. The extraction of natural resources, chemical synthesis, and recombinant expression systems are the three important technologies [17]. Among these, heterologous expression is considered better due to its affordability and effectiveness.

Blue swimming crab or flower crab, *Portunus pelagicus* is an economically important species in tropical and subtropical oceans [18, 19]. Typically, the shallow bays with sandy bottoms are home to huge populations of this species [20]. Crustaceans lack complex and highly specific adaptive immune system like vertebrates and their defense mainly rely on innate immune responses [3]. AMPs play an important role in the innate defense mechanism of crabs like crustaceans. So, the identification and characterization of novel AMPs from *P. pelagicus* would be useful for health management in crustacean culture systems.

In the present study, ALF from *P. pelagicus* was cloned and characterized. Even though the recombinant expression of *P. pelagicus* ALF was tried in *Escherichia coli*, the peptide was found to be lethal to the prokaryotic host *E*. *coli*. A eukaryotic expression system would be a probable solution to this problem.

## Methods

### Experimental organism and hemolymph collection

Live and healthy *Portunus pelagicus* collected from the backwaters of Cochin, Kerala, India, were used for the study. Hemolymph was collected from the base of abdominal appendages using specifically designed capillary tubes (RNase-free) rinsed using pre-cooled anticoagulant −10% sodium citrate, pH 7.0. The collected hemolymph was transferred into TRI reagent (Sigma-Aldrich) and stored at −80°C.

### Total RNA isolation and reverse transcription

The total RNA was isolated from *P. pelagicus* hemolymph using TRI reagent followed by the manufacturer’s protocol. The quantity and quality of the isolated total RNA were checked spectrophotometrically (A_260_:A_280_) and 0.8% agarose gel electrophoresis, respectively. The first-strand cDNA was generated in a total of 20μL reaction mixture with 5μg total RNA, 1X RT buffer, 2mM dNTPs, 2μM oligo d (T_20_), 20U of RNase inhibitor, and 100U of M-MLV Reverse transcriptase. The reaction was carried out for 1h at 42°C followed by an inactivation step at 85°C for 15 min. To check the quality of RNA, an internal control gene *β* actin (forward- 5′ CTTGTGGTTGACAATGGCTCCG-3′; Reverse- 5′ TGGTGAAGGAGTAGCCACGCTC-3′) was used.

### PCR amplification and TA cloning

Amplification of ALF sequence from cDNA of *P. pelagicus* was done using ALF primers (Forward- 5' AGGGAGTGGGTGATGAGCTA 3'; Reverse- 5' TACGGCTATTACGATCCAACA 3'). The reaction volume for PCR was setup by 25μL with 1X standard *Taq* buffer (10 mMTris-HCl, 50 mMKCl, pH 8.3), 3.5 mM MgCl2, 200 μM dNTPs, 0.4 μM each primer, and 1 U *Taq*DNA polymerase (New England Biolabs, USA). The thermal profile for the reaction was as follows; initial denaturation at 95 °C for 2 min followed by 35 cycles of 94 °C for 15 s, 60 °C for 30 s, and 72 °C for 30 s and a final extension at 72 °C for 10 min. Visualization of the amplicons was done by 1.5% agarose gel electrophoresis stained with ethidium bromide using Syngene G: Box Gel documentation unit. The PCR product was purified and cloned in to pGEM®-T Easy cloning vector (Promega). The cloned product was transformed into competent DH5α *E. coli* cells, as per the manufacturer’s protocol. Positive clones were isolated by blue/white screening and confirmed by PCR with gene-specific and vector-specific primers (5′ TGTAATACGA CTCACTATAGGG 3′; Sp6 R (5′ GATTTAGGTGACACTATAG 3′). From the positive clones, single white colony was selected for plasmid isolation. Plasmid DNA was extracted using GenElute™ HP Plasmid Miniprep Kit (Sigma). The isolated plasmid was sequenced with vector-specific primers on an ABI Prism 377 DNA sequencer (Applied Biosystem) at Agrigenome Sequencing Facility, India.

### Molecular characterization of *Pp*-ALF in silico

The nucleotide sequences were assembled and converted into respective amino acid sequences by Expert Protein Analysis System (ExPASy) (http://web.expasy.org/translate/). BLASTn and BLASTp of the National Centre for Biotechnology Information (NCBI) were used for homology searches of nucleotide sequences and deduced amino acids, respectively. The processing site for mature peptide and signal peptide cleavage site were predicted using SignalP 4.1 server (http://www.cbs.dtu.dk/services/SignalP-3.0/). The physico-chemical characterization of the mature peptide was performed with Protparam tool (http://cn.expasy.org/cgi-bin/protparam). Kyte& Doolittle plot in ProtScale tool of ExPASy ((https://web.expasy.org/protscale) was used to analyze the hydrophobicity over the length of the peptide sequence. Amphipathicity and hydrophobic phase of the lipopolysaccharide domain was elucidated by Heliquest online tool (http://heliquest.ipmc.cnrs.fr/cgibin/ComputParamsV2.py).

### Multiple sequence alignment and phylogenetic analysis

Multiple sequence alignment was used for finding conserved domains and motifs of *P. pelagicus* ALF with other ALF sequences retrieved from GenBank. The sequences were aligned by the ClustalW algorithm of BioEdit. The maximum likelihood phylogenetic tree of selected sequences retrieved from NCBI GenBank was constructed with MEGA 7 software based on amino acid sequences. The reliability of the branches was tested using bootstrap resampling with 1000 pseudo-replicates.

### Structural analysis

The RNA sequence (http://www.fr33.net/seqedit.php) of *Pp*-ALF was extracted from the corresponding cDNA sequence and submitted to the RNA structure web server (http://ac.at/cgi-bin/RNAfold.cgi). RNA structure with minimum free energy was predicted by this tool. The secondary structure of the primary amino acid sequence of *P. pelagicus* ALF was elucidated with PSIPRED server ((http://bioinf.cs.ucl.ac.uk/psipred). The spatial structure was constructed by Phyre2 (http://www.sbg.bio.ic.ac.uk/phyre2) algorithm based on homology model templates and viewed by pyMOL software. The Ramachandran plot was constructed by SAVESv6.0 - Structure Validation Server (https://saves.mbi.ucla.edu) to assess the stereochemical quality of the putative protein structure

### Functional analysis in silico

The antimicrobial peptide database (APD3) (http://aps.unmc.edu/AP/main.php) predicted the Wimley-White whole-residue hydrophobicity and Boman index of the peptide. CAMP_R3_ (www.camp3.bicnirrh.res.in), a database of antimicrobial peptide sequences, structures, and signatures, performed sequence optimization prediction with respect to the antibacterial activity of the peptide. The peptide, which is prone to aggregation, was examined with AGGRESCAN (http://bioinf.uab.es/aggrescan/) to determine the short and particular regions of active residues. The tendency of the functional kinds of AMPs was predicted using iAMPpred (http://cabgrid.res.in:8080/amppred/index.html). Anticp (webs.iiitd.edu.in/raghava/anticp/) server computed the probability of the peptide to be an anticancer peptide and antiangiopred (http://crdd.osdd.net/raghava/antiangiopred/) predicted the angiogenesis inhibition property of the peptide. The immunomedicine group (http://imed.med.ucm.es/Tools/antigenic.html) predicted the antigenic property of the peptide. Anti-inflammatory and anti-tubercular properties of the peptide were predicted by AIPpred (http://www.thegleelab.org/AIPpred/) and AntiTbpred (http://thegleelab.org/AtbPpred), respectively. The dPABBs webserver (http://ab-openlab.csir.res.in/abp/antibiofilm/) predicted biofilm inhibitory properties of the peptide. The hemolytic activity of *Pp*-ALF was analyzed by HemoPred (http://codes.bio/hemopred/) and cell-penetrating ability of the peptide was checked by CellPPD server (http://crdd.osdd.net/raghava/cellppd/). DNA-protein interaction-based prediction was evaluated by DNAbinder (http://crdd.osdd.net/raghava/dnabinder/). The immunomodulatory property of peptides was examined by VaxinPAD (https://webs.iiitd.edu.in/raghava/vaxinpad/team.php) prediction portal. PIP-EL (http://www.thegleelab.org/PIP-EL/) prediction server for pro-inflammatory inducing peptides was used to predict the chance of *Pp*-ALF as a PIP.

### Recombinant expression of *P. pelagicus* ALF

The mature peptide sequences of *P. pelagicus* ALF were amplified using primers designed with *BamH*1and *EcoR*1 restriction sites. The Amplicons after purification was inserted into pGEMT^R^–T easy cloning vector and digested with respective restriction enzymes (RE). pET 32a (+) was used as the expression vector. The RE-digested products were gel purified, and the PCR product was inserted into pET-32a (+) plasmid. This transformation produced the recombinant pET-32a-*Pp* ALF which was then sequenced with T7F (5′TGTAATACGACTCACTATAGGG3′) and T7R (5′CTAGTTATTGCTCAGCGGTG3′) primers. The pET-32a-*Pp* ALF plasmid was then transformed into the *E. coli* Rosetta-gami^TM^B (DE3) p Lys S (Novagen, 231 Cricklewood Broadway, London, UK), the expression host. The amplification was confirmed by T7F and T7R primers. The parent vector without an insert fragment was selected as a negative control. After sequencing to ensure in-frame insertion, positive transformants and the negative control were incubated overnight in 10 ml Luria Bertani (LB) medium [containing ampicillin (50 mg/ml), kanamycin (15 mg/ml), chloramphenicol (12.5 mg/ml), and tetracycline (34 mg/ml)] at 37 °C at 250 rpm in an Incubator Shaker (JeioTech, Korea). This was added as inoculum into 250 ml LB medium with the above mentioned antibiotics and incubated at 37 °C at 250 rpm. When the culture reached OD_600_ of 0.5-0.7, IPTG (isopropyl-β-D-thiogalactosidase) was added to a final concentration of 1 mM and incubated. Two ml aliquots of the cultures were taken every hour after induction, centrifuged, and the cell pellet was kept at −20 °C for the detection of the recombinant peptide by 15% SDS-PAGE examination.

### Cytotoxic effect of the peptide *Pp*-ALF on the expression host *E. coli* in the production medium (live/dead assay)

#### SYTO 9–propidium iodide (PI) staining

Since the turbidity of the production medium decreased post-induction and the peptide production could not be detected by SDS- PAGE, the viability of the host cells was checked by SYTO9 - PI staining. The assay was used to analyze the live (membrane intact)/dead (membrane compromised) condition of the expression host *E. coli* Rosetta-gami, due to the production of the peptide *Pp*-ALF. *E. coli* transformed with the pET-32a (+) vector without insert was kept as a negative control, which expresses thioredoxin (Trx). The dye suspension (SYTO 9: PI; 1:1) was added to the cell pellets collected at 0, 1, 2, 4, and 6^th^-hour post-induction, incubated for 15 min in dark at room temperature and observed under Epifluorescence Microscope ( Carl Zeiss Axio observer) with Ex/Em 480/500 nm for SYTO 9 and 490/635 nm for PI.

#### Triple staining (CTC-DAPI-FITC)

In order to observe the cells, both live/dead and membrane permeabilization caused by the peptide, if any in the expression host, triple-staining was done [21]. Fluorochromes used were (i) the double-stranded DNA-binding dye DAPI (stain all bacterial cells both live/dead), (ii) the vital dye CTC (stain only viable cells), and (iii) FITC that traverse only permeabilized cytoplasmic membrane. Both CTC and DAPI were made up as stock solutions in water at a concentration of 6 mg/ml (20 mM) and 10 mg/ml (28 mM), respectively. After induction with IPTG, the host cells (Rosetta-gami pET-32a-*Pp*-ALF) were sampled at 0, 2, and 6 and incubated with 900 μl of CTC for 90 min at 37 ^°^C. An aliquot (10 μl) of the reaction mix was transferred to a poly L-lysine-coated glass slide. The slides were washed with sodium phosphate buffer, and 1ml of DAPI solution (10 μg/ml in PBS) was added and after 30 min at 30 ^°^C. The DAPI solution was removed and the slides were rinsed again with sodium phosphate buffer. To the cells, FITC was added (6 mg/ml in sodium phosphate buffer) and incubated at 30 ^°^C for 30 min and washed as mentioned above. The slides were then examined under a Fluorescence microscope (Carl Zeiss, Germany).

## Results

### Molecular characteristics of *Pp*-ALF

**Fig. 1:**
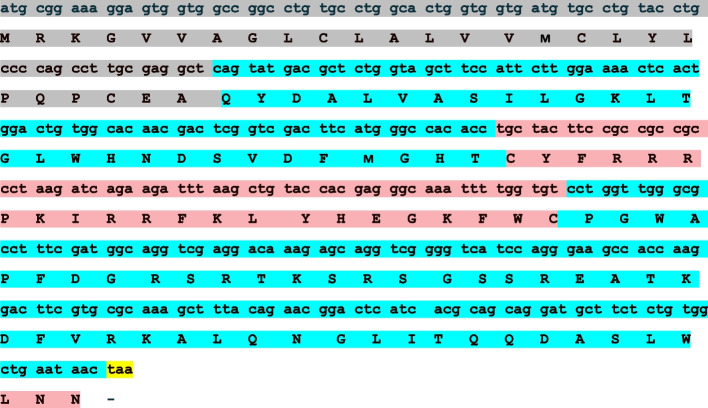
Nucleotide and deduced amino acid sequences of *P. pelagicus* ALF, *Pp*-ALF (OP009359). The single-letter amino acid code is show below the corresponding nucleotide sequence. The gray highlighted region represents the signal peptide, blue represents the mature region, and red indicates the LPS domain

A 369bp nucleotide sequence coding the complete cDNA sequence of ALF encoding 123 amino acids (Fig. 1) obtained from mRNA of *P. pelagicus* hemocyte by reverse transcription. Hereafter, the ALF is referred to as *Pp*-ALF (GenBank ID: OP009359). A similarity search using BLASTn and BLASTp algorithms confirmed the cDNA sequence coming under the ALF family of antimicrobial peptides. Upon BLASTp homology search *Pp*-ALF showed 95.89 % similarity with *Portunus trituberculatus* ALF (GenBank ID: ADU25043.1) followed by *Scylla paramamosain* ALF (GenBank ID: AFI43796.1) with 89.04% similarity. The putative signal peptide was identified at the N-terminal sequence, defined by SignalP software, with the cleavage site between amino acid positions 26 and 27 (CEA-QY). The signal peptide is followed by a highly cationic 97 amino acid mature peptide sequence comprising 22 amino acid long lipopolysaccharide (LPS) binding domain.

**Fig. 2:**
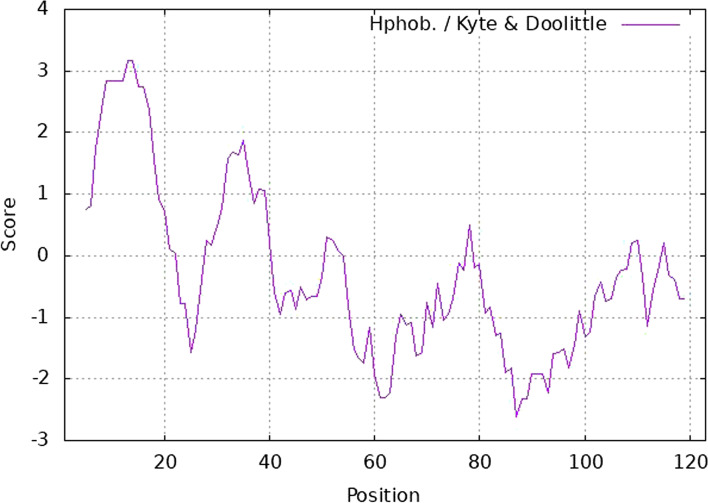
Kyte-Doolittle plot showing hydrophobicity of *Pp*-ALF. The peaks above the score of 0.0 indicate the hydrophobic nature of the peptide

**Fig. 3:**
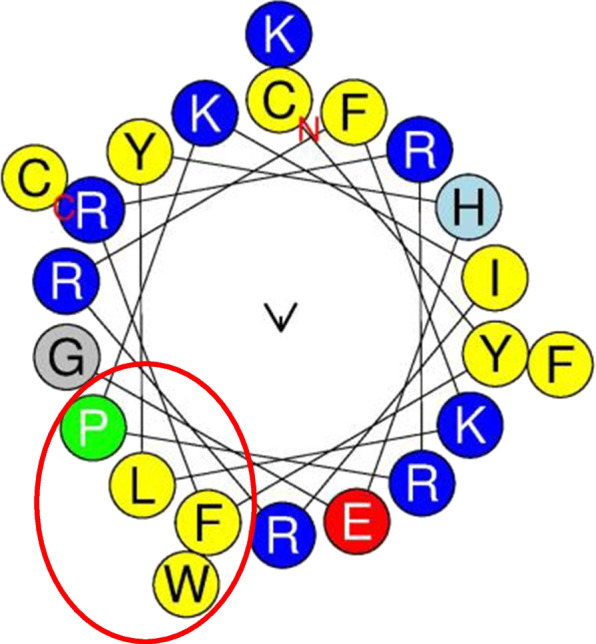
Helical wheel diagram of *Pp*-ALF LPS domain predicted by HeliQuest. The amino and carboxy ends are denoted as N and C, respectively. The predicted hydrophobic face (-FWLP-) was marked with a circle

*Pp*-ALF was found to have a predicted molecular weight of 14.053 kDa, a net charge of +10.75 and a theoretical isoelectric point (p*I*) of 9.8. The mature region of the sequence has aforesaid parameters as 11.29 kDa, +9.75, and 10.17, respectively. The cationic nature of the *Pp*-ALF was mainly contributed by Lys (7%), Arg (9%), and His (2%) against negatively charged amino acids like Glu (3%) and Asp (6%). The predicted half-life of *Pp*-ALF in mammalian reticulocytes is about 30h, in vitro and >10h in *E. coli*, in vivo. The grand average hydropathy value (GRAVY) of the peptide was estimated to be −0.29 and the Wimley white whole residue hydrophobicity was 12.87. Kyte & Doolittle plot revealed the hydrophobicity; the residues from 5 to 21 (-VVAGLCLALVVMCLYLP**-**) in the signal region exhibited the highest hydrophobicity (Fig. 2) which shows the importance of the signal peptide domain for the protein translocation. Residues 28 to 40 (-YDALVASILGKLT**-**) showed the highest hydrophobicity in the mature domain. The amino acid distribution resulting in amphipathicity is illustrated by the helical wheel diagram (Fig. 3). Polar residues including glycine made up 50% of the predicted total, whereas non-polar residues made up the remaining 50%. The peptide’s hydrophobicity was 0.378 H, with a hydrophobic moment of 0.060μH and the hydrophobic phase is supplied by -FWLP- residues.

### Multiple sequence alignment and phylogenetic analysis

**Fig. 4:**
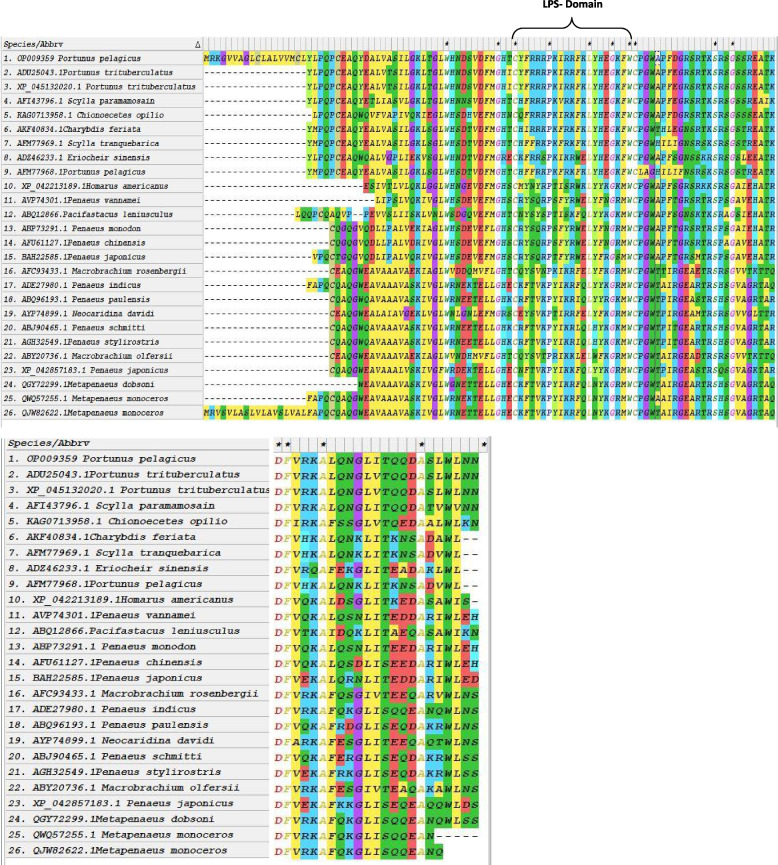
1 & 2 Clustal W multiple alignments of *P. pelagicus* ALF, *Pp*-ALF with other reported crustacean ALFs using MEGA version 7. LPS domain is shown in the bracket and marked

**Fig. 5:**
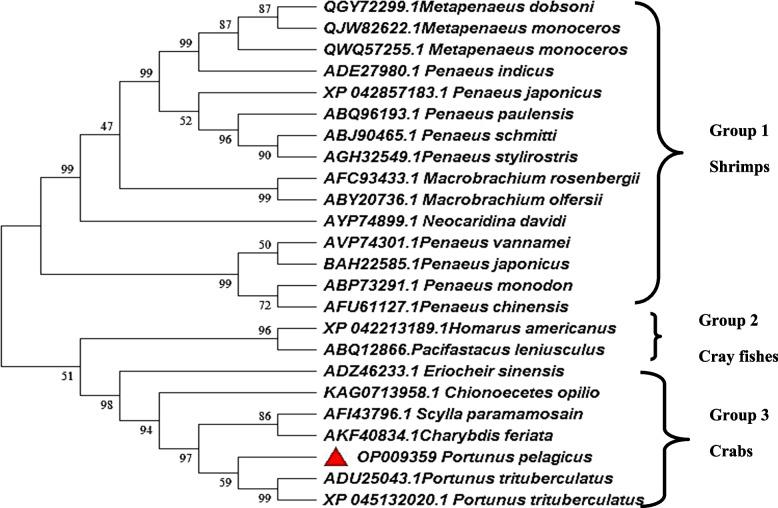
Maximum likelihood tree obtained using MEGA 7 showing the phylogenetic relationship of *Pp*-ALF with other crustacean ALFs

The multiple sequence alignments of *Pp*-ALF with other crustacean ALFs retrieved from GenBank revealed the conserved motifs and residues. The two cysteine residues (Cys^55^-Cys^76^) involved in the internal disulfide bond formation were totally conserved in *Pp*-ALF (Fig. 4). *Pp*-ALF possessed the highest similarity with *P. trituberculatus* (ADU25043.1) (95.89%) in terms of amino acid composition. *Pp*-ALF has Ala at the 33rd position, which is substituted by Thr in *P. trituberculatus*, similarly, Thr is substituted with Ile at the 54th position. A consensus pattern of WCPGWA (T) was also observed in all ALFs. The evolutionary relationships with other crustacean lineages were found by phylogenetic analysis. Bootstrapped method using deduced amino acid sequences was used to elucidate the ancestral relationships (Fig. 5). The tree forms three groups, group 1 includes ALFs from shrimps, group 2 comprises ALF from cray fishes, and group 3 consists of crab ALFs including the *Pp*-ALF from *P. pelagicus*.

### Structural characteristics

**Fig. 6:**
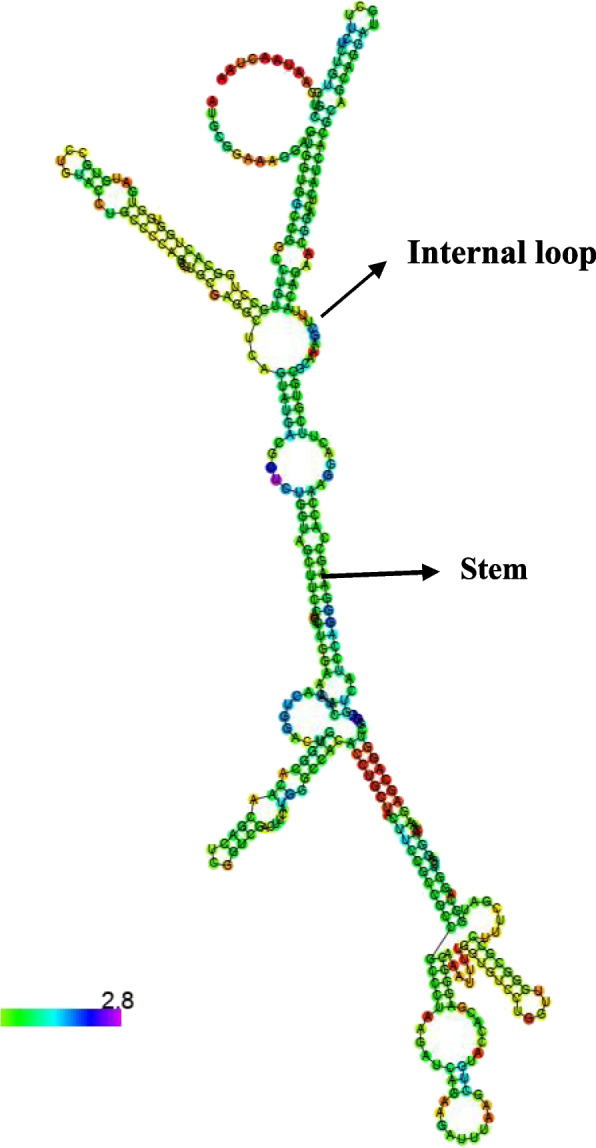
mRNA structure of *Pp-*ALF showing stem-loop structure. The mRNA structure is colored by base pairing probabilities. A high base-pairing probability is indicated by the red color and a low base-pair probability is indicated by the blue color

**Fig. 7:**
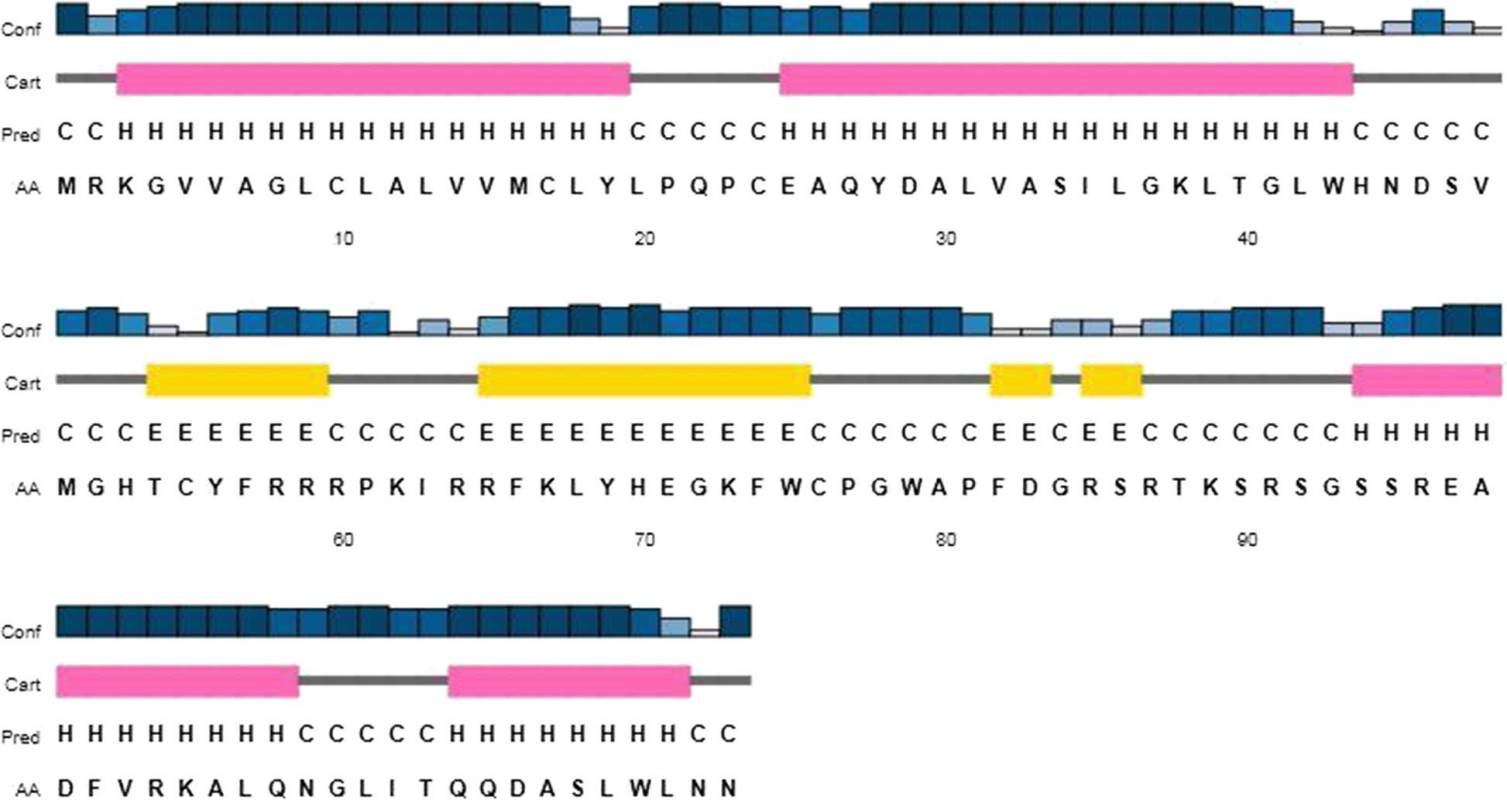
Secondary structure of *Pp-*ALF peptide predicted using PSIPRED server. The alpha-helical structure is represented by the pink color, beta-strand by the yellow color, and the coils by the gray color, respectively

**Fig. 8:**
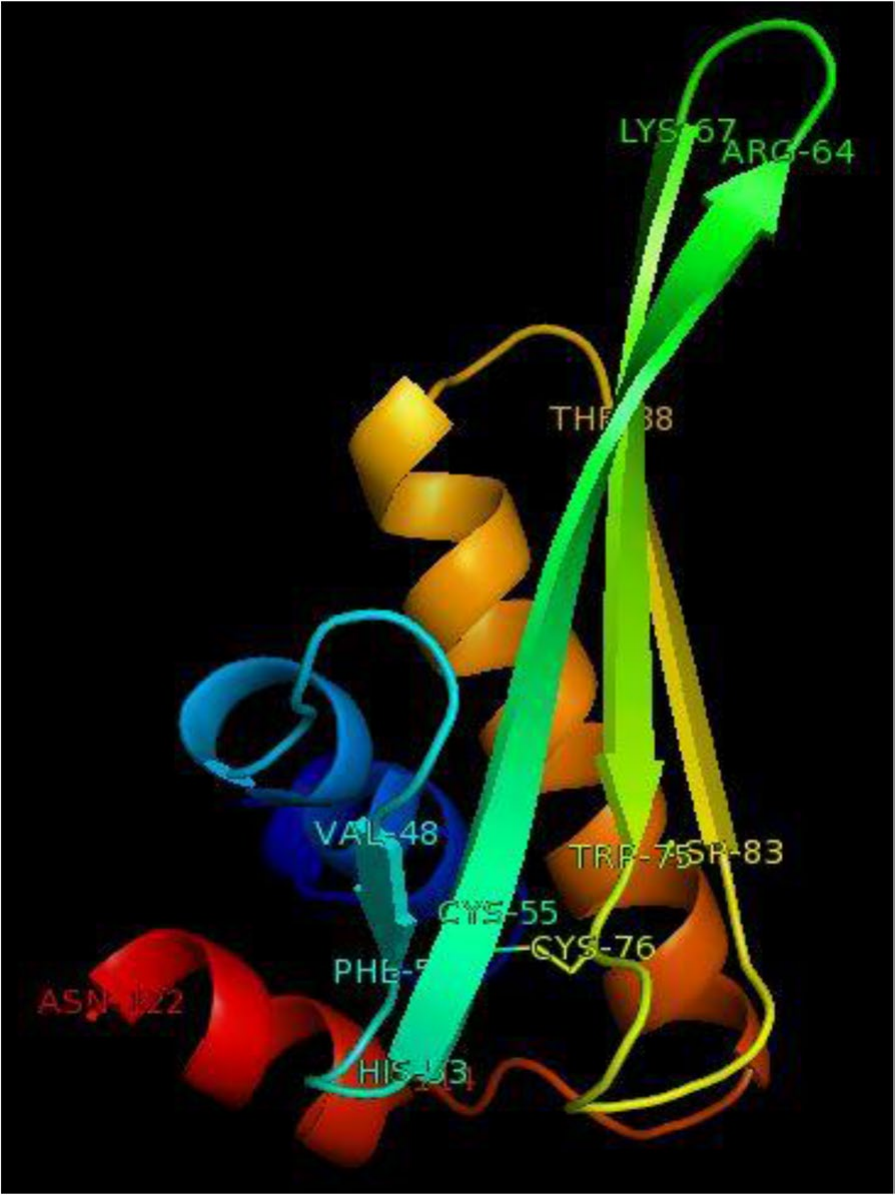
The diagrammatic representation of the 3D structure of *Pp-*ALF drawn with PyMOL software using the PDB data generated by Phyre2 software

**Fig. 9:**
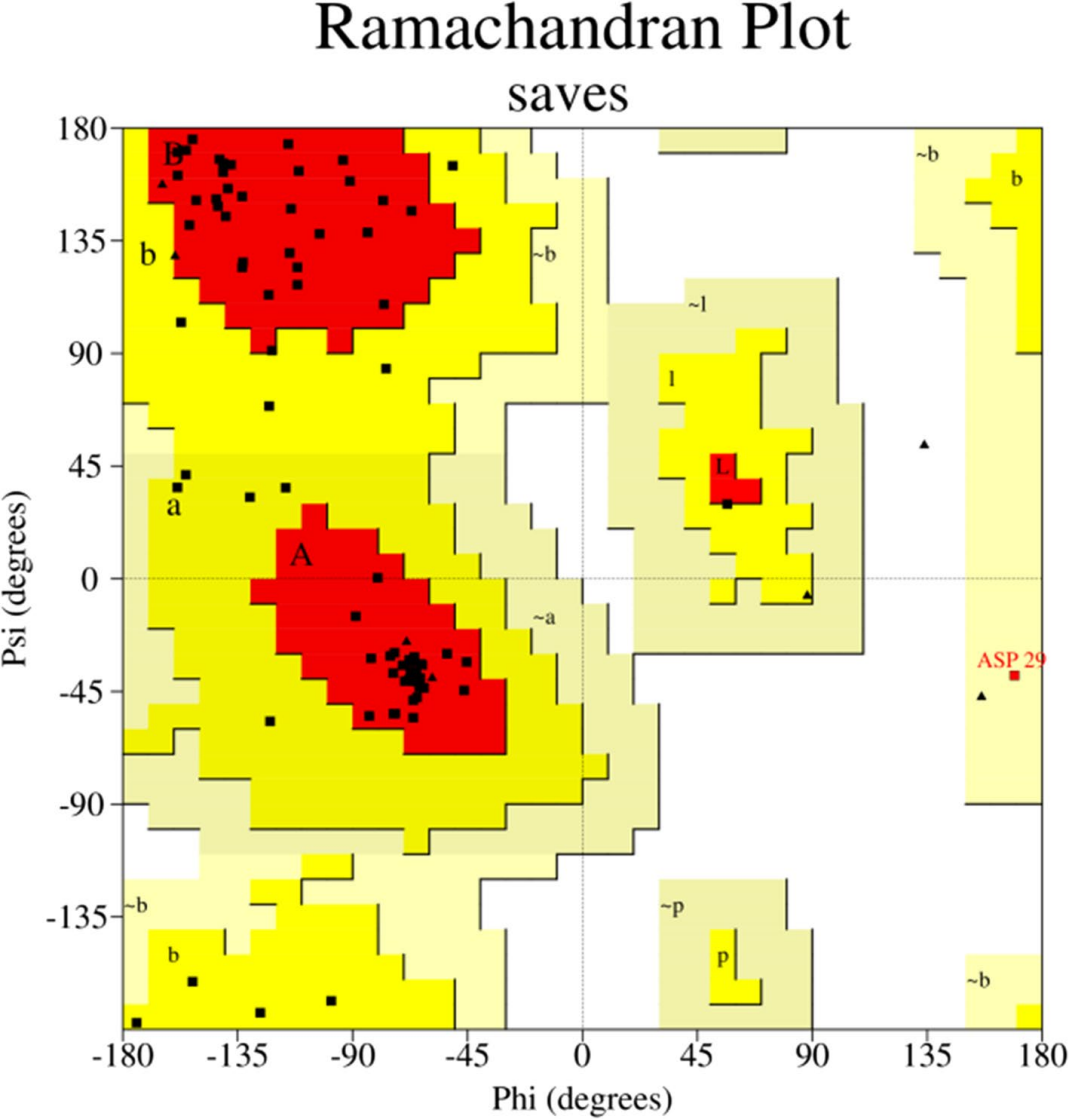
Ramachandran plot for the predicted a three-dimensional structure of *Pp-*ALF using PROCHECK server

*Pp*-ALF had a predicted mRNA structure with minimum free energy (MFE) of −123.30 kcal/mol. This indicates the structural stability of the stem-loop structure of mRNA (Fig. 6). The nucleotides were mostly paired and formed intramolecular base pairing of RNA. The secondary structure of *Pp*-ALF showed an *α* helical structure in N-terminal signal peptide region followed by the other two helices towards C-terminal of the mature region. Between these helices consists of segments of *β* strands and randomly coiled structure. The amphipathic LPS binding domain was formed by *β* strands and coiled segments (Fig. 7). The spatial orientation of the *Pp*-ALF was constructed using the solution structure of an ALF (c2jobA) as template. The template shared 100% confidence with *Pp*-ALF. The 3D structure (Fig. 8) consists of three *α* helices packed against four *β* sheets. The *β* sheets, 1 (His ^53^-Arg ^64^) and 2 (Lys ^67^-Trp ^75^) are linked by a conserved disulphide bond (Cys^55^-Cys ^76^) forming the highly cationic (+7.25, p*I* 10.54) and amphipathic *β* hairpin loop structure, the LPS binding domain. The highly cationic LPS domain permits the peptide to effectively interact with negatively charged bacterial membranes. Structure validation of *Pp*-ALF by Ramachandran plot (Fig. 9) showed 85.2% residues in the most allowed region, 13.6% in the additionally allowed area, and 1.1% amino acid residues in the generously allowed region. No residues were found in the disallowed region. Based on the analysis, the R-factor is no greater than 20% and a good-quality model is expected to have over 90% residues in the allowed regions.

### Functional characteristics

CAMP server predicted *Pp*-ALF as an antimicrobial peptide using support vector machine (SVM) classifier. *i*AMPpred identified *Pp*-ALF as antibacterial, antiviral, and antifungal with 0.96, 0.96, and 0.98 SVM score, respectively. SVM score near to 1 is identified as a peptide with high therapeutic potential. Even though *Pp*-ALF was predicted to exhibit antimicrobial potential, the region with high aggregation propensity towards microbial membrane was predicted by AGGRESCAN. There are two hotspots predicted for *Pp*-ALF; the first hotspot (HS) area with a normalized value of 0.745 was found inside the signal peptide domain (GVVAGLCLALVVMCLYL), and the second HS was inside the mature region (DALVASILGKLTGL) with a normalized value of 0.347.

The LPS domain of *Pp*-ALF was predicted with anti-angiogenic activity by “AntiAngioPred” with an SVM score of 0.82 indicating its anticancer property. Also, the average antigenic propensity of *Pp*-ALF was found as 1.0366 by the “Immunomedicine group.” The server predicted 5 antigenic sequence regions in *Pp*-ALF, i.e., 4 to 38 residues (GVVAGLCLALVVMCLYLPQPCEAQYDALVASILGK), 49 to 57 (DFMGHTCYF), 64 to 70 (RRFKLYH), 72 to78 (GKFWCPG), and 112 to119 (ITQQDASL). So, *Pp*-ALF could be used as an immunogen with prediction values of 0.316 (AntiTbpred) and 0.574 (AIPpred) as anti-tubercular and anti-inflammatory peptide. Biofilms provide survival sites for opportunistic pathogens. The LPS domain of *Pp*-ALF was predicted as an antibiofilm agent (SVM score −0.83). The ability to distinguish between an antigenic cell and host cell is an important property of an ideal AMP. HemoPred predicted the LPS domain of *Pp*-AF as non-hemolytic. This property leads to the safe application of the peptide in the biological system.

Peptides are divided into two types based on their amino acid composition; cell-penetrating and non-cell-penetrating peptides. *Pp*-ALF was predicted to be CPP (cell-penetrating peptide) with a positive SVM score of 0.01. CPPs are made up of basic amino acids like arginine and lysine, and they can cross membranes to obtain access to the cell’s core. *Pp*-ALF consists of a total of 17 arginine and lysine residues in the mature region of the peptide. This was confirmed by “DNA binder”, which gave the *Pp*-ALF a positive score of 1.25, indicating it as a DNA-binding protein. *Pp*-ALF possesses immunomodulatory activity as predicted by “VaxinPAD” with an SVM value of 0.97. So, *Pp*-ALF has the potential to stimulate the innate immune system as an adjuvant. In immunotherapy, pro-inflammatory peptides (PIP) have been employed as anticancer agents, antibacterial agents, and vaccines. *Pp*-ALF has been predicted as pro-inflammatory peptide (PIP) with a probability score of 0.7633. The projected value of the codon adaptation index, which represents the likely success of heterologous gene expression was, 0.57 in *Escherichia coli*, 0.58 in *Saccharomyces cerevisiae*, and 0.6 in *Pichia pastoris*. The peptide rank score was calculated to be 0.99, indicating the high bioactive potential of *Pp*-ALF.

### Recombinant expression of* Pp*-ALF and cytotoxicity to the expression host

#### Live/dead assay by SYTO 9-PI staining of cells

**Fig. 10:**
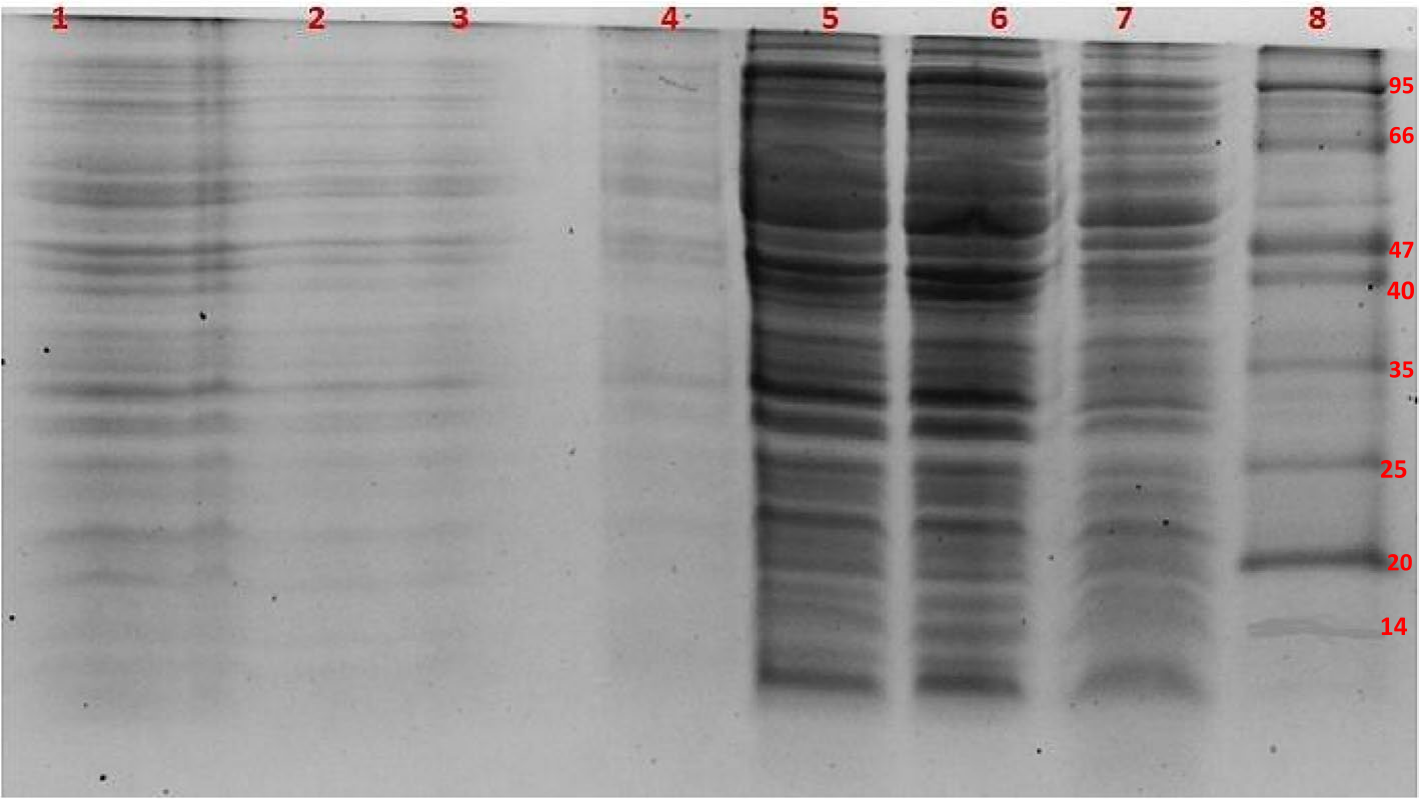
Tricine SDS-PAGE analysis of the *E. coli* cells with pET-32a-*Pp*-ALF before and after IPTG induction on a time-course basis. Lane 1: Uninduced control (before IPTG induction). Lane 2–7 IPTG-induced cells after 0–6h of induction lane 8: Mid-range protein marker

**Fig. 11:**
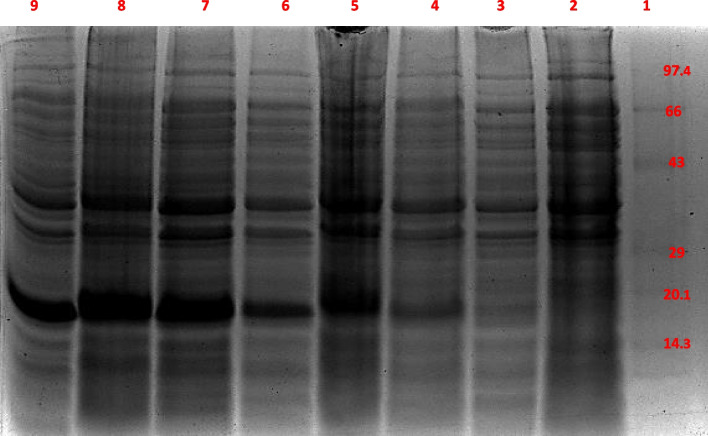
Tricine SDS-PAGE analysis of the cells containing recombinantly expressed Thioredoxin tag (negative control), before and after IPTG induction on a time-course basis. Lane 1: Mid-range protein ladder; Lane 2: Uninduced control (before IPTG induction); Lanes 3–9: IPTG-induced cells after 0–6h of induction

Turbidity of the production medium with the expression host *E. coli* (pET-32a- *Pp*-ALF) was found decreasing post-induction with IPTG. The cell lysate of the expression host *E. coli* when analyzed by SDS-PAGE did not display the presence of the recombinant peptide *Pp*-ALF (29.08kDa with Trx tag) (Fig. 10). However, thioredoxin (Trx 17.72 kDa) could be detected in control (expression host *E. coli* with pET-32a) (Fig. 11).

**Fig. 12:**
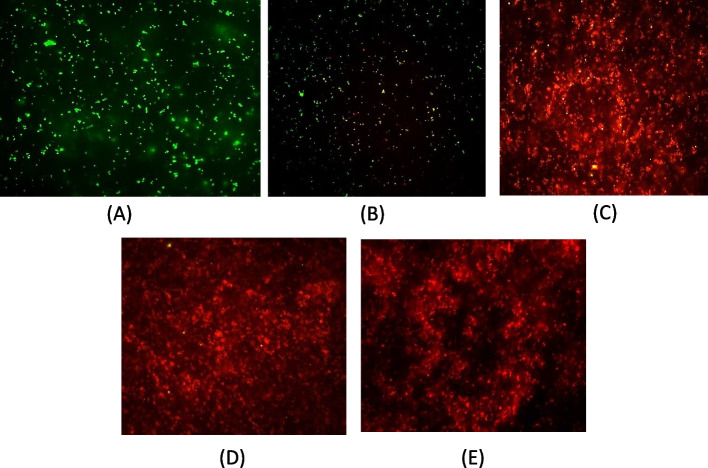
SYTO 9-PI staining of expression host, *E.coli* after IPTG induction during recombinant production of *Pp*-ALF **A** 0^th^ h, **B** 1^st^ h, **C** 2^nd^ h, **D** 4^th^ h, and **E** 6^th^ h

**Fig. 13:**
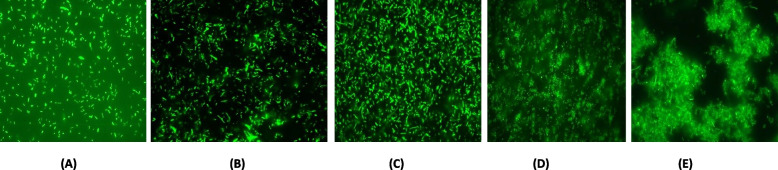
SYTO 9-PI staining of expression host, *E.coli* after IPTG induction of negative control: **A** 0^th^ h, **B** 1^st^ h, **C** 2^nd^ h, **D** 4^th^ h, and **E** 6^th^ h

SYTO 9 - propidium iodide (PI) staining of the host cells revealed the death of the cells during the production of *Pp*-ALF. The recombinantly expressed *Pp*-ALF causes the death of the expression host, and this was observed on staining. The cells were green fluorescent at the 0^th^ hour since they were metabolically active. From the 1^st^ hour post-induction itself, the cytotoxic effect on host cells was clearly visible and from the 2^nd^ hour onwards complete cell death was confirmed by red fluorescence due to the internalization of propidium iodide stain (Figs. 12 and 13). Apart from this, the bacteria form a mucilaginous sheath in response to the peptide production. In the case of control, cells were active throughout the production period (5 h post-induction).

### Live/dead/membrane permeabilizationby CTC-DAPI-FITC staining

**Fig. 14:**
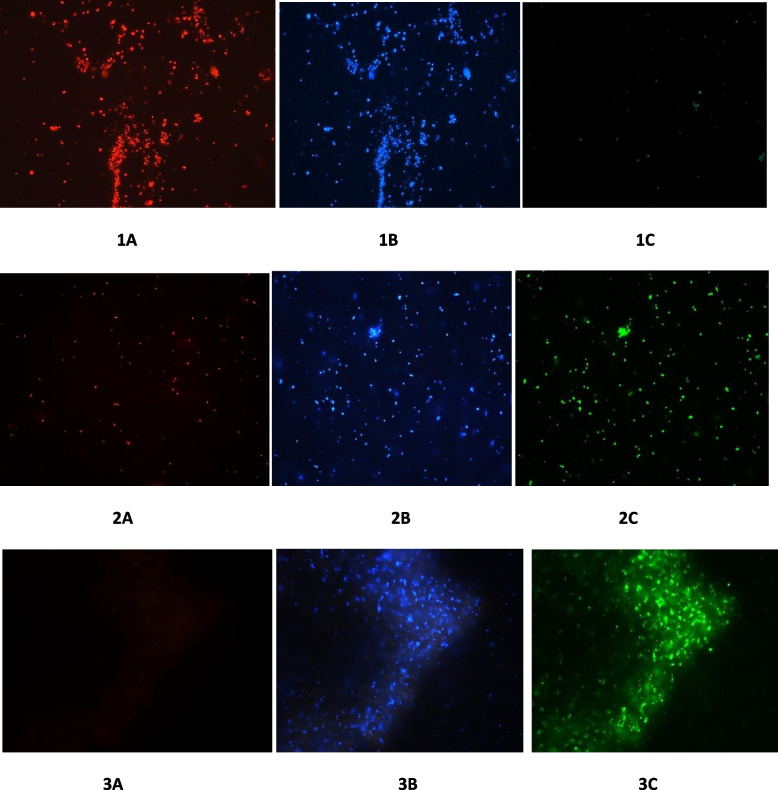
Triple staining images of expression host, *E. coli* during recombinant expression of *Pp*-ALF 1A-C: 0^th^ h- CTC, DAPI, and FITC-stained cells 2A–C: 2^nd^ h 3A–C: 6^th^ h

Cell death as well as permeabilization was not noticed initially (at the 0^th^ h) at the time of IPTG induction as evidenced and confirmed by CTC-DAPI–FITC staining. Reduction in viable cells and membrane permeabilization was observed at the 2^nd^ h. Viable cells were not observed at the 6^th^ h, and only membrane-permeabilized/dead cells were detected. This concludes that the peptide *Pp*-ALF causes death to the host cells at the initial stages of production itself. The peptide also causes a high degree of cellular clumping which was also observed in cells at the 6^th^ h (Fig. 14).

## Discussion

Due to the emergence of antibiotic-resistant microbes, the significance of alternate compounds like AMPs to combat infections has been increased [22]. In the present study, an anti-lipopolysaccharide factor *Pp*-ALF was cloned from *P. pelagicus.* An essential physicochemical property of AMPs is their hydrophobicity [23–26]. *Pp*-ALF possesses a consensus pattern of “WCPGWT”, besides the highly conserved pair of cysteine residues and the LPS-binding domain [15, 27]. The newly discovered *Pp*-ALF has three helices packed against four β sheets, which is similar to the 3D structure of LALF (Limulus ALF), ALF*Pm3*, (*Penaeus mondon*) *Sp*ALF4 (*Scylla paramamosain*), and *Sp*ALF7 [28–31]. The disulfide bond is crucial for the stability of the 3D structure of ALF [29]. On phylogenetic analysis, the ALF sequences were found clustering into species-specific groups. The ALFs from crabs, shrimps, and crayfishes were all grouped independently. A further finding from the data is that *Pp*-ALF has a stronger association with crab ALFs than shrimp and crayfish ALFs.

The LPS-binding domain of *Pp*-ALF possesses 8 hydrophobic amino acids including one tryptophan. It has been demonstrated that the ALFs with high p*I* value, bind with LPS inhibiting the proliferation of Gram-negative bacteria [32–34]. Inside the amphipathic loop of LPS in *Pp*-ALF, nine positively charged amino acid residues are present with a high p*I* of 10.54. Rosa et al. [35] reported that ALF with higher isoelectric point possesses high antibacterial activity. In *S. paramamosain*, SpALF1 and SpALF2 with high p*I* showed antimicrobial activity against both Gram-negative and Gram-positive bacteria [30].

The biological functions of ALFs mainly depend on the LPS domain that interacts with the negatively charged LPS [36]. Positively charged amino acids (arginine and lysine) in the LPS domain contribute to the antibacterial activity of the organisms and the activities were found to diminish, if the arginine and lysine residues were substituted with neutral amino acids [5, 37, 38]. Substitution of lysine in a parotid secretory protein, GL13NH2 changed its functional property from bacterial agglutinating peptide to bactericidal peptide [39]. Due to the high prevalence of lysine and/or arginine in most AMPs, their net charges range from +2 to +11 [40, 41]. *Pp*-ALF had a net charge of +10.17 for the mature peptide indicating the high cationicity of the peptide. It is generally accepted that the initial interaction of the AMP with the negatively charged membrane surface of the bacterium is predominantly caused by cationicity [42, 43]. LPS domain of *Pp*-ALF embedded with five arginine and three lysine residues contributing 23 and 14% of the total amino acids, respectively, in the LPS region indicating its potential role as an antimicrobial agent. FcALF2 (*Fenneropenaeus chinensis* ALF) and PmALF3 (*Penaeus monodon* ALF) with five and six positively charged amino acids in the LPS domain have shown strong antibacterial activity [35, 44]. *Pp*-ALF shares important characteristics of LALFs (Limulus ALF), with the hydrophobic N-terminal sequences and the concentration of positive charges in the disulphide loop that are crucial for their antimicrobial effect [45, 46].

*Pp*-ALF was found to possess 41% hydrophobicity as per APD analysis. Hydrophobicity controls the degree to which a peptide can partition into the lipid bilayers and therefore play an important role in its activity [47–49]. Hydrophobicity is necessary for membrane permeabilization and higher hydrophobicity results in higher antibacterial action [23, 50]. The hydrophobicity of sSpALF7 from *Scylla paramamosain* was higher (51.4%) than that of rSpALF7 (41.5%), which might be one of the reasons why sSpALF7 showed a broader antibacterial spectrum. Analysis of the *Pp*-ALF sequence using the Kyte-Doolittle plot exhibited a significant presence of hydrophobic amino acids concentrated in the first 17 residues (-VVAGLCLALVVMCLYLP-**)** of the mature peptide. This demonstrates that these amino acids are components of the alpha helix and may have the ability to traverse a lipid bilayer of the microbial membrane [51]. *Pp*-ALF was predicted to possess antibacterial, antifungal, and antiviral activities, and it was found to have high therapeutic potential according to the prediction score. Six recombinant PtALF proteins could prevent the growth of specific Gram-positive, Gram-negative bacteria, or fungi in the swimming crab *P. trituberculatus* [34, 52].

ALF from *P. monodon* showed inhibition against herpes simplex virus type 1, human adenovirus respiratory strain, and WSSV replication [16, 53]. LBD peptides, i.e., FcALF1, FcALF2, FcALF5, and ALFFc from *Fenneropenaeus chinensis* could inhibit WSSV replication [44]. Crab-ALF2A and crab-ALF6A, isolated from *P. trituberculatus*, showed minimal effective concentrations (MECs) of 2.11 μg/mL and 1.95 μg/mL against the yeast *Candida albicans*, respectively, demonstrating promising antiviral and antifungal activity against yeasts and viruses [13]. ALFPm3 from black tiger shrimp *P. monodon* [54] showed antifungal and antibacterial activities. Bacterial cell membranes have been destroyed by mFcALF2 (modified ALF from *Fenneropenaeus chinensis*) resulting in the leakage of cytoplasm [55]. MjALF-D from *Marsupenaeus japonicas* also exhibited antibacterial activity [4]. *Pp*-ALF is predicted as a cell-penetrating peptide and therefore might cause pore formation resulting in the leakage of cell contents.

Synthetic SALF (shrimp ALF) from black tiger shrimp displayed anticancer properties in HeLa cells when tested in mice. SALF disrupted the tumor cell membrane triggering apoptosis [56]. *Pp*-ALF is also predicted to have antiangiogenic properties by the Anticp and antiangiopred server with a significant prediction score.

The majority of the studies have concentrated on the production of AMPs in bacteria due to the availability of well-defined plasmid vectors and the practical significance of large-scale peptide production through fermentation. However, since some of these peptides are toxic to the expression host, direct expression of AMPs is typically challenging [57]. As a result, these peptides are produced as fusion proteins in bacterial strains lacking proteases, including *E. coli* BL21. The fusion approach enhances solubility and prevents toxicity and cell disintegration in the expression host. The recombinant expression has greater benefits than high-cost chemical synthesis and low-yield natural extraction. Due to the potent antibacterial properties displayed by *Pp*-ALF in silico, recombinant production was attempted using the *E. coli* expression system. However, the peptide was found to be toxic to the host cells since post induction with IPTG, the cells started dyeing which might be due to the production of the peptide *Pp*-ALF. rMnALF4 (recombinant ALF from *Macrobrachium nipponense*) also showed toxicity in *E. coli* expression system; so it was produced in *P. pastoris* with a high yield. Additionally, *Salmonella typhi*, *Shigella* sp., *Staphylococcus aureus*, *Pseudmonas fluorescens*, and *Salmonella gullinarum*, all of which are extremely pathogenic for humans, were sensitive to rMnALF4 [17]. *Pp*-ALF production was toxic to the host, which was proved by live dead SYTO 9-PI staining and triple staining. Death of the expression host was observed from the first hour onwards after IPTG induction.

The eukaryotic yeast expression host, *P. pastoris* is widely used in the heterologous expression of antimicrobial peptides, because of its potent secretion and glycosylation property. Anti-lipopolysaccharide factors from *P. mondon* [58], *Macrobrachium rosenbergii* [59], *Macrobrachium nipponense* [17], and *Litopenaeus vannamei* [60] were successfully expressed in the yeast expression system, *P. pastoris*. ALF from *M. nipponense* could not be expressed in *E. coli* [17], and in the present study also, *Pp*-ALF could not be expressed in *E. coli*. The reason for this differential antibacterial effect displayed by *E. coli* could not be explained.

Recombinant proteins can take on specific spatial structures and post-translational modifications when they are expressed by the eukaryotic expression system. These changes would better replicate the recombinant proteins’ natural state in living organisms, and the structure and modification are crucial for protein function [31]. For the synthesis of AMPs, especially those with significant bacterial inhibitory action, the prokaryotic expression system, such as the *E. coli* system, is not typically used [59, 61]. Although the precise processes by which AMPs exert their microbicidal action are not yet fully understood, it is generally agreed that AMPs primarily target the cytoplasmic membrane through penetration and cell lysis activities [62]. Conditional toxicity is the main issue in this situation; specifically, the AMP should not be toxic to the expression host while it is forming, but should be active when employed to combat the infecting bacterium [63]. Recombinantly generated peptides lack this property and are also vulnerable to enzymatic digestion and lack post-translational modification. Recombinant proteins can be successfully synthesized as secretory proteins in the yeast *P. pastoris* expression system when a signal peptide is linked to the foreign protein at its N-terminus [64]. Since *Pp*-ALF was toxic to *E. coli* cells, an alternate expression system like *P. pastoris* would be a viable option*.* The success of heterologous expression predicted by the codon adaptation index showed that *P. pastoris* system has the highest chance for expression of *Pp*-ALF*.* Synthetic biology is also another option for the synthesis of these molecules [65]. The use of bioactive peptides in the development of novel medical therapies would be highly promising. This work provides concise information with regard to an AMP, anti-lipopolysaccharide factor characterized from *P. pelagicus* with potent antimicrobial activity predicted in silico and displayed by the death of the host *E. coli* DH5 alpha cells on heterologous expression.

## Conclusion

Functional prediction of the anti-lipopolysaccharide factor from *P. pelagicus*, *Pp*-ALF revealed that it could be a potent molecule with respect to antimicrobial, anti-inflammatory, and anticancer activity. The prokaryotic expression system *E. coli* was not found suitable for the recombinant expression of *Pp*-ALF due to its cytotoxic effect on the host cells. Eukaryotic expression systems would be a preferred option for the recombinant production of *Pp*-ALF.

## Data Availability

The data generated during and/or analyzed during the current study are not publicly available (except GenBank accession) but are available from the corresponding author on reasonable request.

## Abbreviations

AMP: Antimicrobial peptides
ALF: Anti-lipopolysaccharide factor
*Pp*: *Portunus pelagicus*
